# Microvascular Alterations in Alzheimer's Disease

**DOI:** 10.3389/fncel.2020.618986

**Published:** 2021-01-18

**Authors:** Joe Steinman, Hong-Shuo Sun, Zhong-Ping Feng

**Affiliations:** ^1^Department of Physiology, University of Toronto, Toronto, ON, Canada; ^2^Department of Surgery, University of Toronto, Toronto, ON, Canada

**Keywords:** Alzheimer's disease, amyloid hypotheses, vasculature, microvasculature, TRPM2, angiogeneis, growth factors, imaging

## Abstract

Alzheimer's disease (AD) is a neurodegenerative disorder associated with continual decline in cognition and ability to perform routine functions such as remembering familiar places or understanding speech. For decades, amyloid beta (Aβ) was viewed as the driver of AD, triggering neurodegenerative processes such as inflammation and formation of neurofibrillary tangles (NFTs). This approach has not yielded therapeutics that cure the disease or significant improvements in long-term cognition through removal of plaques and Aβ oligomers. Some researchers propose alternate mechanisms that drive AD or act in conjunction with amyloid to promote neurodegeneration. This review summarizes the status of AD research and examines research directions including and beyond Aβ, such as tau, inflammation, and protein clearance mechanisms. The effect of aging on microvasculature is highlighted, including its contribution to reduced blood flow that impairs cognition. Microvascular alterations observed in AD are outlined, emphasizing imaging studies of capillary malfunction. The review concludes with a discussion of two therapies to protect tissue without directly targeting Aβ for removal: (1) administration of growth factors to promote vascular recovery in AD; (2) inhibiting activity of a calcium-permeable ion channels to reduce microglial activation and restore cerebral vascular function.

## Preface

Many drugs developed to treat Alzheimer's disease (AD) have been designed on the assumption that amyloid beta (Aβ) drives the disease. This approach has not yielded FDA-approved drugs, with approved medications such as cholinesterase inhibitors or memantine (glutamate blocker) only treating symptoms of AD. Proponents of the Amyloid Hypothesis (AH) feel that anti-Aβ therapies may have been administered too late in the disease process, and that the AH remains valid. To others, disease mechanisms beyond Aβ accumulation must be targeted.

Movement beyond traditional interpretations of the AH requires understanding the myriad of processes altered in AD. This influences research direction and treatment development. This review summarizes the status of AD research, and includes the examination and discussion of multiple mechanisms underlying AD pathology. Alternative hypotheses of AD beyond the AH, such as abnormal tau and inflammation, are detailed. Microvascular alterations in aging and AD are discussed, and their role assessed in reducing blood flow and impairing Aβ clearance from tissue. Techniques and methods for imaging microvasculature in AD are outlined, with results from studies highlighting the role of capillary imaging and mathematical analysis in understanding vascular contributions to AD. The review concludes by discussing potential therapies that promote vascular recovery and protection against Aβ toxicity without targeting Aβ.

## Introduction: An Overview

AD is associated with decline in episodic memory, impaired concentration or attention, disorientation, and loss of verbal fluency (Vandenberghe and Tournoy, 2005; Ferris and Farlow, 2013). Examples of factors contributing to loss of cognition include age-related capillary reductions and blood brain barrier (BBB) leakage (Farkas and Luiten, 2001; Zlokovic, 2005), release of pro-inflammatory cytokines from microglia that induce neuron death (Floden et al., 2005; Wang et al., 2015), plaque-induced neural network disruption (Knowles et al., 1999; Kuchibhotla et al., 2008), and toxic soluble Aβ oligomers that cause synaptic dysfunction (Smith and Strittmatter, 2017).

The AH proposes that extracellular Aβ deposition initiates neurodegenerative processes, such as neurofibrillary tangle (NFT) formation, leading to cognitive impairment (Hardy and Higgins, 1992; Makin, 2018). It was initially believed that elimination of plaques via targeting Aβ would improve cognition. This was supported by promising results in transgenic PDAPP mice overexpressing mutant human APP, where immunization with Aβ prevented plaque development and neurite damage (Schenk et al., 1999).

Current treatments, such as cholinesterase inhibitors, temporarily improve symptoms such as memory loss and reasoning capacity, without altering Aβ deposition or tau pathology (Govindpani et al., 2019; Madav et al., 2019). By passage of 3 months, the effect of these treatments is reduced due to tolerance development in patients (Madav et al., 2019).

One possibility for developing AD treatments is targeting multiple therapeutic sites in addition to, or separately from, Aβ (Selkoe, 2019). This could enable use of approved medications developed to treat other conditions. For example, a recent study that applied etodolac (a nonsteroidal anti-inflammatory) to an *in vitro* AD cell culture model found enhanced BBB integrity and reduced cell death in response to neurotoxins (thrombin) administered into the endothelial cell barrier (Shin et al., 2019).

Targeting multiple therapeutic sites in addition to Aβ requires understanding the complexity of AD pathology and numerous changes that occur possibly independent of Aβ. For example, BBB dysfunction may occur prior to Aβ deposition in individuals possessing the apolipoprotein E4 gene (ApoE4), where the ApoE4 gene is known to render susceptibility to AD (Montagne et al., 2020). Tau, a microtubule associated protein linked to synaptic loss and dysfunction, is more closely related to cognitive impairment than amyloid (Bejanin et al., 2017). Susceptibility to AD may be dependent on gender, with women twice as likely as men to contact AD. This is possibly due to metabolic alterations during menopause (Ferretti et al., 2018). Microglia, central nervous system (CNS) immune cells, may operate protectively by surrounding amyloid deposits, compacting fibrils into a less toxic form and preventing plaque growth. They may also harm neurons through release of inflammatory mediators (Hansen et al., 2018). This dual role highlights the complexity of the tissue response to AD.

Often overlooked in Alzheimer's research is the microvasculature, which is commonly viewed as secondary to neuronal death and inflammation. Recent microscopy studies suggest a critical role of the microvasculature in disease progression. Cruz Hernández et al. (2019) demonstrated via vascular and neutrophil imaging with 2-photon microscopy in AD mice and mathematical blood flow modeling (in mouse, human, and synthetic microvascular networks) that capillary flow blockage via neutrophils reduces cerebral blood flow (CBF). CBF was restored through antibody targeting of neutrophils, which improved short-term memory function up to 16-months of age in APP/PS1 mice (Bracko et al., 2020). Nortley et al. (2019) demonstrated capillary compression by pericytes induced by amyloid-triggered release of endothelin could lead to widespread reductions in blood flow. These findings suggest the microvasculature could contribute to damage of brain cells and synapses (Nortley et al., 2019).

Consideration of aspects of the disease beyond amyloid are necessary for the development of therapies. The following section discusses the AH and presents alternatives. Microvascular changes and their contributions to AD follow. The review concludes by discussing two therapies that do not target Aβ directly: (1) vascular endothelial growth factor (VEGF) administration to improve vascular survival (Religa et al., 2013); (2) inhibiting the calcium-permeable ion channel transient receptor potential melastatin 2 (TRPM2) to protect against Aβ toxicity (Ostapchenko et al., 2015).

## Alzheimer's Research: The Amyloid Hypothesis

### Alzheimer's Research: A General Summary

Although the AH influenced and directed drug development for many years, there has been a shift in opinion toward exploring alternatives. Up to 2019, ~22% of clinical trials tested amyloid therapies, while significant research efforts are focused on a range of targets such as neurotransmitters and tau (Liu et al., 2019).

Promising results have been obtained with anti-Aβ strategies. Aducanumab, a human monoclonal antibody directed against Aβ, reduced soluble and insoluble parenchymal Aβ in Tg2576 AD mice, while reducing Aβ in humans and slowing clinical decline (Sevigny et al., 2016). However, Aducanumab provided insufficient relief from functional and cognitive decline in two Phase 3 clinical trials, though subsequent analyses indicated reduced rate of behavioral and cognitive decline (Walsh and Selkoe, 2020). Another antibody, BAN2401 cleared amyloid plaques and slowed cognitive decline in a Phase 2b study (Walsh and Selkoe, 2020).

Despite the promise of these anti-Aβ therapeutics, it is unclear whether removal of amyloid by itself would “cure” AD. For example, widespread inflammation may be initiated by Aβ deposition, limiting the benefits of amyloid removal if inflammation is not simultaneously reduced. Aging reduces capillary density and blood flow, which may contribute to cognitive deficits alongside amyloid. According to the Mitochondrial Cascade Hypothesis, genetically, and environmentally determined decline in mitochondrial function triggers AD pathology (Swerdlow et al., 2014). In this instance, Aβ removal may only marginally improve AD (Swerdlow et al., 2014). Some researchers contend neural network function is a better predictor of disease progression when compared with plaques (Kosik, 2013). These disparate views indicate a lack of consensus in treating or detecting AD and could be attributable to the wide variety of mechanisms which initiate AD and aid its progression. Genetic risks associated with AD may be related to APP production and processing, as well as other processes such as the immune system and neuroinflammation (Bertram and Tanzi, 2019). A single intervention targeting Aβ removal may therefore be unable to significantly reverse disease progression.

### The Amyloid Hypothesis

APP is a transmembrane protein processed by enzymes β- and γ-secretase in the amyloidogenic pathway (Stakos et al., 2020). The length of the Aβ peptide produced by this cleavage is 38–42 amino acids, depending on the position where γ-secretase cleaves APP. It is largely the 42-amino acid peptide that aggregates into soluble oligomers, ultimately coalescing into extracellular plaques.

According to the AH, overproduction of APP or failure of clearance mechanisms causes amyloid accumulation (Mucke, 2009). As Aβ concentration increases, oligomers are formed, followed by plaques. Oligomers alter cell signaling and trigger release of toxic molecules by microglia. Oligomers and dimers prevent glutamate reuptake by astrocytes and neurons, leading to excitotoxicity, while plaques distort and damage axons and dendrites (Mucke, 2009; Selkoe, 2019; Zott et al., 2019). Tangle formation is due to Aβ activation of kinases catalyzing tau phosphorylation, neuroinflammation and cytokine release, reduced ability to degrade tau, and Aβ-induced impaired axonal transport (Blurton-Jones and Laferla, 2006; Silva et al., 2019).

Genetic evidence supports the AH. Mutations in three genes, *APP, PSEN1*, and *PSEN2*, contribute to early-onset AD. The *APP* gene encodes the amyloid precursor protein, while *PSEN1* and *PSEN2* encode presenilins (Lanoiselée et al., 2017). Genetic variants influencing Aβ and APP processing are also associated with later-onset dementia (dementia after 65) (Kunkle et al., 2019).

Mice models which overexpress APP develop plaques and memory impairment, but not NFTs. This may be due to absence of human tau in some mouse models (Selkoe and Hardy, 2016). Choi et al. (2014) demonstrated in human neural cell culture that mutations in *APP* and *PSEN1* produced extracellular amyloid plaques and tau pathology. Amyloid and tau were reduced through administration of β- or γ-secretase inhibitors, indicating that targeting Aβ peptide generation reduces plaques and tangles (Choi et al., 2014).

Further evidence in favor of the AH is described in Walsh and Selkoe (2020) and Selkoe and Hardy (2016). In Down's Syndrome an additional copy of Chromosome 21 produces an extra APP gene, causing the individual to display AD-like neuropathology. This is followed by microgliosis (proliferation and migration of microglia), astrocytosis (astrocyte proliferation in response to brain damage), and NFTs (Selkoe and Hardy, 2016). In contrast, mutations which reduce amyloid deposition protect against AD (Selkoe and Hardy, 2016). In general, early inherited AD is caused by APP mutations or in the proteins generating APP. These mutations, and corresponding biomarkers such as CSF Aβ concentration, may precede other pathologies including tau deposits (this may be disputed, see below), tissue atrophy, glucose hypometabolism, and cognitive decline by many years (Walsh and Selkoe, 2020). The apolipoprotein E4 (ApoE4) gene is expressed in more than 50 % of AD patients, and is the most common AD genetic risk factor (Safieh et al., 2019). ApoE4 increases Aβ production via its effect on γ-secretase activity, impairing lysosomal Aβ degradation, and possessing impaired ability to transport Aβ across the BBB (Safieh et al., 2019).

In contrast to the AH, some studies suggest Aβ is not the sole cause of AD. Tau correlates more strongly than amyloid with cognitive impairment (Bejanin et al., 2017). While genetic factors for late-onset AD include those affecting Aβ, others affect pathways related to tau binding proteins or alternate mechanisms (Kunkle et al., 2019). Subtle cognitive deficits may occur prior to or coincident with early amyloid build-up, suggesting AD may be initiated prior to amyloid pathology (Thomas et al., 2020). A counterargument in support of the AH is Aβ deposition may be an early event that initiates AD pathology and cognitive decline (Selkoe and Hardy, 2016). Aβ build-up may not be expected to correlate with cognitive performance in this interpretation.

Active immunization with Aβ prevents amyloid deposition in mouse models, although effectiveness of treatments is diminished in mice with extensive amyloid deposition (Das et al., 2001). Immunization causes mice to generate antibodies through Aβ exposure, enabling microglial to bind to and eliminate plaques. This therapy translated to humans reduced amyloid burden, however cognitive improvement was absent. Some patients demonstrated signs of severe dementia despite clearance of amyloid deposits (Holmes et al., 2008; Gallardo and Holtzman, 2017). Furthermore, 6% of immunized patients developed meningoencephalitis (Gilman et al., 2005).

Amyloid treatments may not be successful is amyloid removal via therapy causes amyloid to deposit on vessel walls, inducing hemorrhage and trapping toxic peptides within tissue in mice and humans (Wilcock et al., 2004; Patton et al., 2006). Another possibility is soluble Aβ surrounds the plaques and prevent the administered antibody from reaching the plaques in sufficient concentration (Demattos et al., 2012).

Failures have also occurred with passive immunotherapies where an externally produced antibody is administered, as opposed to one internally generated through immunization. For example, bapineuzumab failed to reduce amyloid load or phosphorylated tau in cerebrospinal fluid (CSF) in phase 3 trials (Vandenberghe et al., 2016).

Several factors may contribute to failure of AH-based drugs beyond a simple assumption the AH is flawed. Amyloid deposition occurs many years prior to cognitive decline (Jack et al., 2013). Therapies may be administered by the time the effects of neuronal loss and tissue atrophy are irrevocable. This may be overcome in clinical trials using biomarkers to select patients with mild dementia attributable to early AD (Frisoni and Blennow, 2013). Selection of appropriate biomarkers to detect early AD or monitor progress is complex since numerous biomarkers are applicable, such as positron emission tomography (PET) amyloid imaging, fluorodeoxyglucose PET to detect hypometabolism, CSF tau, MRI to detect brain atrophy, and CSF YKL-40 (an indicator of activated microglia) (Zetterberg and Bendlin, 2020).

In humans abnormally phosphorylated tau occurs in nerve cells or their processes prior to 30 years of age, and before extracellular amyloid plaques deposits (Braak and Del Tredici, 2011). Phosphorylated tau could spread to other brain regions, such as the brainstem and entorhinal cortex, contributing to AD (Jack et al., 2013). This appears to support the Tau Hypothesis (tau drives AD progression, see Section The Tau Hypothesis: Tau as a Driver of Neurodegeneration), suggesting that targeting amyloid may be ineffective if abnormal tau is not concurrently eliminated. An alternative interpretation is since tauopathy occurs in asymptomatic individuals, tauopathy may be an element of aging but not AD specifically (Jack et al., 2013). Tauopathy and Aβ pathology may occur independently, with tauopathy preceding Aβ deposition. Aβ accelerates tauopathy, eventually leading to NFTs in the neocortex (Musiek and Holtzman, 2012; Jack et al., 2013). This potentially occurs in mice, where breeding APP-transgenic with tau-transgenic mice increases tau deposition, but does not alter Aβ (Lewis et al., 2001; Selkoe and Hardy, 2016).

Aβ antibodies may not clear soluble oligomers, which are believed to more significantly influence synapse loss and cognition compared to plaques. Plaques serve as “reservoirs” of soluble and toxic Aβ, without being as directly toxic themselves (Koffie et al., 2009; Walsh and Selkoe, 2020). This characteristic may partially explain why some individuals with plaques do not appear to suffer cognitive consequences, since these cognitively normal individuals have a low ratio of soluble oligomer to plaque ratios (Walsh and Selkoe, 2020).

Challenges with drug therapies may not be due to Aβ specifically, but drug design. γ-secretase inhibitors potentially may inhibit Aβ accumulation by preventing cleavage at the γ-secretase site. Many γ-secretase inhibitors have been identified, however they are not specific for cleavage of APP. They also inhibit processing of Notch and other proteins involved in development, cell adhesion, hematopoiesis, and contacts between cells (Evin et al., 2006). This may induce serious side effects. Treatment with a γ-secretase inhibitor, semagacestat, was associated with cognitive decline and skin cancer as a side-effect. This skin cancer was potentially attributable to blocking Notch1 cleavage (Extance, 2010).

### The Tau Hypothesis: Tau as a Driver of Neurodegeneration

In the Tau Hypothesis, tau (a microtubule-associated protein) replicates and spreads between cells. This causes neurodegeneration through mechanisms such as synaptic impairment and changes to mitochondrial structure and function through interactions between tau and actin (DuBoff et al., 2012; Kametani and Hasegawa, 2018). Phosphorylated tau induces cognitive deficits through decreasing the number of synapses and triggering cell death (Di et al., 2016).

In a PET study, the intensity of tau signal, but not Aβ, predicted the rate of tissue atrophy at early clinical AD stages (La Joie et al., 2020). Tau pathology could appear in healthy older adults without amyloid (Harrison et al., 2019), and may be present in brains of individuals with mild dementia absent Aβ pathology (de Paula et al., 2009). Extensive tau pathology is believed to occur as a result of Aβ accumulation, as demonstrated in neuronal cell cultures, mouse models, and genetic studies (Selkoe, 2011; Choi et al., 2014). This renders it unlikely to initiate AD by itself, although tau abnormalities may begin in childhood (Braak and Del Tredici, 2011).

Drugs targeting tau phosphorylation or aggregation have not successfully translated from mice to humans. For example, glycogen synthase kinase-3 (GSK3β) is a serine/threonine kinase facilitating tau hyperphosphorylation. In a mouse model overexpressing human mutant APP and tau, a GSK3β inhibitor affected multiple targets including reduced tau phosphorylation and amyloid deposition. This potentially protected against neuronal death and memory deficits through inhibiting the intrinsic mitochondrial signaling pathway to reduce cellular damage (Serenó et al., 2009). Clinical benefits were not obtained when a GSK-3 inhibitor, Tideglusib, was tested in clinical trials (Lovestone et al., 2015). Similar to Aβ therapies, possibly clinical trials of GSK-3 inhibitors are conducted at too late a disease stage to be effective (Hernandez et al., 2013).

### Inflammation

In early AD, scavenger receptors (SRs) on microglia promote Aβ clearance (Wang et al., 2015). As amyloid builds, continual microglial interaction with Aβ via additional receptors (such as CD36) cause release of pro-inflammatory cytokines, leading to neuronal damage (Wang et al., 2015).

AD brains demonstrate chronic inflammation, with microglia displaying increased activity in AD patients (Liu et al., 2019). Amoeboid microglia indicating an activated inflammatory state are associated with Aβ, with microglial activation occurring following amyloidosis and neuronal injury, and possibly promoting tau accumulation (Suárez-Calvet et al., 2016; Hemonnot et al., 2019). Microglia may damage neurons directly as evident in axonal damage co-localizing with microglial cells *in vitro* (Park et al., 2018).

Inflammation may be a causative factor in AD. Epidemiology studies indicate a possible protective effective of anti-inflammatories (Andersen et al., 1995). Ibuprofen reduced amyloid deposition, cognitive decline, and inflammatory markers in transgenic mice (Lim et al., 2000; Van Dam et al., 2010). In wild type mice, a systemic prenatal immune challenge caused AD-like pathology, with elevation in inflammatory cytokines, increased hippocampal APP, and altered tau phosphorylation (Krstic et al., 2012).

A genetic risk associated with plaque clearance in AD is CD33 expression, a myeloid cell receptor expressed by microglia activated by glycoproteins and glycolipids and displayed on plaques (Hollingworth et al., 2011; Naj et al., 2011; Zhao, 2019). CD33 expression is elevated in AD, and deletion of the gene in the APP/PS1 mouse model reduces insoluble Aβ and plaque levels (Griciuc et al., 2013). The activated receptor recruits proteins such as SHP phosphatases that inhibit phagocytosis (Zhao, 2019). CD33 inhibitors are an area of drug development research, although translation from mice to humans is challenging due to species differences in protein domain structure (Biber et al., 2019). Another transmembrane receptor found in microglia and neurons associated with AD is triggering receptor expressed by myeloid cells 2 (TREM2), which is involved in phagocytosis and preventing inflammatory mediator production (Doens and Fernández, 2014). TREM2 mutation reduces microglial clustering around plaques in mice and humans, resulting in enhanced neuritic dystrophy (Carmona et al., 2018).

Scheiblich et al., 2020 use a “Wave Model” to describe inflammation progression in AD. Pattern recognition receptors (PRRs) on microglia detect Aβ, followed by tau and other misfolded proteins. This activates proinflammatory pathways and toxic cytokines contributing to AD such as TNFα, IFNγ, ROS, and IL (interleukin)-1β, 4, 6, 9, 12, 23 (Mandrekar-Colucci and Landreth, 2010; Scheiblich et al., 2020). TNFα and IFNγ reduce levels of insulin degrading enzyme, impairing degradation of Aβ, and also impair microglial phagocytosis of Aβ (Mandrekar-Colucci and Landreth, 2010). The interleukins and TNFα trigger neuronal signaling leading to phosphatase inactivation and kinase activation, causing tau hyperphosphorylation, aggregation into toxic forms, and ultimately neuronal loss and dysfunction (Scheiblich et al., 2020). This neuronal loss is associated with altered concentrations of neurotransmitters, and enhanced astroglial and microglial proliferation (Scheiblich et al., 2020). An element of the inflammatory response is the NLRP3 inflammasome, which activates caspase-1 and ASC specks, which then bind to Aβ and induce its aggregation (Venegas et al., 2017; Scheiblich et al., 2020). The ASC specks may be absorbed by myeloid cells, resulting in sustained immunoactivity (Venegas et al., 2017).

Inflammation exerts effects on blood vessels through perivascular cells. In healthy tissue, perivascular macrophages (PVMs, macrophages contacting a vessel or within a cell thickness of a vessel) limit vessel permeability through maintenance of endothelial cell tight junctions, phagocytose pathogens, restrict inflammation, and control leukocyte movement across the vasculature (Lapenna et al., 2018). PVMs have a protective role by scavenging amyloid, as seen in a study in TgCRND8 mice where depletion of PVMs enhanced CAA load (Hawkes and McLaurin, 2009; Lapenna et al., 2018). However, Aβ stimulates PVM ROS release, contributing to cerebrovascular oxidative stress and vascular dysfunction through smooth muscle contraction (Lapenna et al., 2018). In wild-type mice where Aβ_40_ was infused through the carotid artery, PVM depletion restores the ability of the vasculature to increase blood flow in response to functional stimulation or vasodilators (Park et al., 2017). The source of PVM-induced vascular dysfunction is Aβ in bloodstream that crosses the vascular wall, binding to CD36 (cell surface receptor) on PVMs and leading to Nox2 ROS production (Park et al., 2017; Lapenna et al., 2018).

### Additional Factors Influencing Alzheimer's Progression

#### The Female Gender

Most AD patients are female (~2/3), with females exhibiting reduced glucose metabolism in comparison with males (Mosconi et al., 2018). Menopause is believed to increase AD risk. Estrogen has protective effects such as reducing neuronal vulnerability to apoptosis through expression of anti-apoptotic proteins, and reducing oxidative stress through acting on mitochondria (Henderson and Brinton, 2010). Studies in 3xTg AD mice demonstrated ovariectomy reduced memory performance and increased Aβ accumulation (Carroll et al., 2007). Hormone replacement therapy has therefore been proposed to reduce AD risk. If administered prior to or at menopause in humans, glucose metabolism is preserved in regions susceptible to AD and prevents cognitive decline, potentially through increasing blood flow (Maki and Resnick, 2000; Brinton, 2008; Rasgon et al., 2014; Scheyer et al., 2018). This therapy is ineffective in postmenopausal women and is associated with an increased risk of AD (Savolainen-Peltonen et al., 2019). Long-term estrogen depletion may render an individual non-responsive to therapy due to decreased hippocampal estrogen-receptor-α (Scheyer et al., 2018). Alternatively, damage to mitochondria may cause estrogen to increase neurological damage (Brinton, 2008; Scheyer et al., 2018).

#### Abnormal Protein Disposal Mechanisms

Late onset AD is characterized by impaired Aβ clearance (Mawuenyega et al., 2010). Most Aβ is cleared through the BBB, although cerebrospinal fluid (CSF) contributes to Aβ clearance (Ramanathan et al., 2015). Subarachnoid CSF enters brain parenchyma through paravascular spaces around penetrating arteries. It mixes with interstitial fluid (ISF) in the brain interstitium, facilitated by aquaporin 4 (AQP4, a water channel protein) in astrocytes. This CSF-ISF mixture, via bulk flow or diffusion, travels through the tissue and is eliminated along paravascular spaces surrounding veins (Iliff et al., 2012; Reeves et al., 2020). The ISF bulk flow between these two pathways (i.e., para-arterial and paravenous routes) clears Aβ from the brain. Since ISF flow is mediated by AQP4 in cell membranes of astroglia, and due to its similarity to the lymphatic system, this pathway is termed the “glymphatic system” (Iliff et al., 2012). Ultimately, solutes (such as Aβ) and ISF diffuse to subarachnoid CSF, enter the bloodstream, or travel along the venular walls to the lymphatic system (Iliff et al., 2012). The importance of the glymphatic pathway is seen in the study by Xu et al. (2015), where AQP4 deletion in 12-month old APP/PS1 mice increased Aβ and cerebral amyloid angiopathy (CAA), as well as increased atrophy of astrocytes and memory impairment.

This fluid movement is driven largely by cardiac-generated penetrating arterial pulsation. This was shown in a mouse study where reducing pulsatility through internal carotid artery ligation reduces arterial pulsation and CSF-ISF exchange (Iliff et al., 2013). In a mathematical model, it was demonstrated that decreasing heart rate (and consequently arterial pulsations) and increasing arterial stiffness led to elevated brain Aβ deposition (Kyrtsos and Baras, 2015).

Plaque build-up along vessels contributes to reduced amyloid clearance. CAA increases arterial wall stiffness, reducing pulsatility and ISF movement (Reeves et al., 2020). Aβ deposition may also reduce space around arteries for fluid flow, increasing accumulation in tissue (Reeves et al., 2020).

Studies do not generally correlate vascular changes, such as microvessel density or tortuosity, with glymphatic clearance. Peng et al. (2016) found suppression of glymphatic transport in both aged (12–13 months) and young (3–4 months) APP/PS1 mice prior to significant Aβ deposits or CAA, when extensive vascular loss begins to occur. Since vessel density in APP/PS1 mice at 18-months does not differ between transgenic and age-matched controls (Hooijmans et al., 2007), it is reasonable to assume that the impaired glymphatic clearance at 3–13 months is not attributable to vessel loss. Mice models do experience changes in microvascular morphology at early time points prior to vascular loss (Durrant et al., 2020). It is conceivable that these morphological changes reflect dysfunctional vasculature, which could contribute to impaired Aβ clearance.

Aging contributes to impaired glymphatic clearance, since older mice experience a drop in penetrating arterial wall pulsatility, with a corresponding reduced ability to clear Aβ injected into brain tissue (Kress et al., 2014). In humans, loss of vascular compliance is associated with Aβ deposition, possibly due to structural changes in elastin (Fonck et al., 2009). Kress et al. (2014) propose that reduced compliance with aging is a contributor to reduced pulsatility. Alternatively, since astrocytes contact arteries via their endfeet, changes to astrocytes may occur during reactive astrogliosis that could affect arterial pulsatility (Kress et al., 2014).

Extracellular proteins may be degraded via cellular secretion of protein-degrading enzymes (Tarasoff-Conway et al., 2015). The two main routes of Aβ clearance in cells is through the ubiquitin-proteasome and autophagy-lysosome pathways (Perez et al., 2014). Due to reduced proteasome activity with aging, Aβ accumulates intracellularly, causing excessive lysosome activity that prevents cells from increasing protein degradation rate in response to stress (Ding et al., 2003; Perez et al., 2014).

#### Environmental Risk Factors

Nitrogen oxides and tobacco smoke are associated with greater dementia risk (Killin et al., 2016). Higher lead concentrations lead to an increased likelihood for AD, possibly attributable to astrocyte activation and alterations to tight junction proteins that induce BBB dysfunction (Killin et al., 2016).

While air particles may enter the body through lungs and blood stream, they commonly enter through the olfactory bulbs. A study (Calderón-Garcidueñas et al., 2010) of Mexico City residents found olfactory bulb inflammation and Aβ_42_ accumulation in vessels, neurons, and glia. A significant contributor to Aβ_42_ accumulation is ultrafine nanoparticles in the environment that promote oligomer formation/fibrillation (Linse et al., 2007; Calderón-Garcidueñas et al., 2010). They are dangerous because they are not bound to membranes, enabling access to organelles, and proteins (Calderón-Garcidueñas et al., 2010).

## Vasculature in Alzheimer's Disease

Vessel damage in AD is present in vessels of all sizes. This includes flow reductions in large arteries in the Circle of Willis (Krucker et al., 2004) and penetrating arterioles displaying a twisted morphology (Dorr et al., 2012). Section Vasculature in Alzheimer's Disease below focuses on capillaries and microvasculature since capillary compression is a significant contributor to reduced CBF, and BBB damage may initiate or precede dementia (Cruz Hernández et al., 2019; Nation et al., 2019; Montagne et al., 2020).

### The Aging Vasculature and Alzheimer's

Reduced capillary density with aging is attributed to diminished levels of angiogenic growth factors (such as VEGF), an imbalance between production of angiogenic and anti-angiogenic growth factors, and reduction of nitric oxide release and impaired vasodilation (Rivard et al., 1999; Ahluwalia et al., 2013; Ambrose, 2017).

Ambrose (2012) proposed the Neuroangiogenesis Hypothesis, wherein a decline in growth factors and angiogenic cytokines leads to a reduction in vessel density and cognition. Restoration of vessel density through administration of growth factors, such as VEGF, is proposed as a treatment to prevent development of AD symptoms (see Section Discussion).

In addition to vessel loss, aging is linked to increased capillary tortuosity and a thickened basement membrane (Østergaard et al., 2015). Pericytes are lost or become dysfunctional, causing BBB dysfunction and impaired flow regulation that decreases oxygen concentration in tissue (Kisler et al., 2017; Berthiaume et al., 2018). Aging may cause downregulation of the endothelial low density lipoprotein receptor 1 (LRP1) due to activation of protein kinase Cα (Silverberg et al., 2010). LRP1 eliminates Aβ proteins through the BBB, and its reduced concentration causes amyloid build-up.

Decreased neprilysin (the main Aβ degrading enzyme) in cells with aging leads to impaired amyloid clearance ability (Sasaguri et al., 2017). Aβ peptides (in particular the more soluble Aβ_40_) therefore comprise a large proportion of atherosclerotic plaques (Kokjohn et al., 2011). Atherosclerosis develops prior to brain Aβ deposition and neural dysfunction in APP23 mice, indicating a possible contribution or connection between atherosclerosis and dementia (Tibolla et al., 2010). Most AD individuals display atherosclerosis in the Circle of Willis, with arteries to which blood is delivered by the Circle of Willis displaying decreased blood flow and increased pulsatility index (Gupta and Iadecola, 2015). Decreased blood flow causes tissue hypoxia and elevated Aβ production through increased β-secretase activity (Gupta and Iadecola, 2015). Increased arterial stiffening, as indicated by the elevated pulsatility index, transfers excessive pulsatile energy to capillaries (Wåhlin and Nyberg, 2019). This energy transfer leads to pericyte injury and BBB breakdown in the hippocampus, impairing memory function (Wåhlin and Nyberg, 2019).

Atherosclerosis and AD are associated with blood flow reductions, vessel occlusions, and arterial wall thickening. Differences exist between the conditions. Atherosclerosis is associated with cholesterol-rich arterial wall deposits, while AD is characterized by plaques and tangles leading to neuron loss. Deposition of Aβ plaques on blood vessels, as in CAA, further reduces blood flow. Whereas in atherosclerosis, many deposits are on major arteries (including outside the brain, such as the aorta), in AD deposits are located on intracerebral arterial vasculature (Lathe et al., 2014), such as penetrating arterioles in TgCRND8 mice (Dorr et al., 2012) and capillaries in a human study (Hecht et al., 2018). Capillary deposits correspond to impaired blood flow in the APP23 mouse model (Thal et al., 2009). In humans, these deposits are associated with tissue microinfarcts in the hippocampus and cognitive decline (Hecht et al., 2018). In atherosclerosis, macrophages laden with cholesterol accumulate in vessel walls, diminishing blood flow to multiple tissues. Rupture of these plaques may lead to stroke or myocardial infarction (Lathe et al., 2014). While atherosclerosis and AD may share similarities, and atherosclerosis is a risk factor for AD, causes of reduced blood flow and tissue damage differ.

Vascular risk factors are prominent in aged populations. This may be due to aging-induced inflammation and cytokine release, leading to endothelial dysfunction and arterial stiffening (Sun, 2015). Hypertension is associated with microvascular abnormalities such as endothelial swelling and reduced capillary density (Boudier, 1999; Østergaard et al., 2015). This microvascular deficiency in hypertension is potentially aggravated by a deficiency in circulating insulin-like growth factor 1 (IGF-1) due to aging (Tarantini et al., 2016). However, a meta-analysis from our lab (ZP Feng) was not able to conclusively determine whether there was an increase or decrease in IGF-1 levels in AD subjects, indicating that IGF-1 may not be the source of microvascular dysfunction in AD (Ostrowski et al., 2016).

### Cerebral Amyloid Angiopathy and the Vascular Hypothesis

In rodents, reductions in CBF increase Aβ deposition and induce memory deficits (Wang et al., 2016). Reduced blood flow may increase amyloid pathology through activation of β/γ-secretases that induces APP cleavage (Cai et al., 2017), through failure of Aβ drainage from the brain (Weller et al., 2008, 2009), or increased rate of tau phosphorylation (Koike et al., 2010). Aβ may deposit on artery walls or leptomeningeal vessels, and to a lesser extent capillaries (Preston et al., 2003; Thal et al., 2008; Weller et al., 2009). This is termed cerebral amyloid angiopathy (CAA), and is associated with vessel bleeding (Thal et al., 2009; Yates et al., 2014).

There are two main CAA subtypes: Type 1 occurs in cortical capillary walls, arterioles, leptomeningeal and cortical arteries, and venules/veins, whereas Type 2 does not occur in capillaries (Thal et al., 2002). Capillary CAA is regarded as a distinct CAA type, occurring in populations with a high ApoE4 allele frequency and correlating with AD pathology severity (Kövari et al., 2013). In arteries and veins, there is a higher ratio of Aβ_40_:Aβ_42_ compared to senile plaques, with a similar ratio in capillaries compared to senile plaques (Thal et al., 2008). Potentially, this higher ratio of Aβ_40_ is due its solubility, enabling it move within perivascular drainage pathways and accumulate in vessel walls (Greenberg et al., 2020). CAA begins as small Aβ deposits on basement membranes of arteries, eventually depositing in the muscle cell layer (Thal et al., 2008). In severe cases, the smooth muscle cell layer deteriorates, and capillaries become occluded in mice and humans, reducing cerebral blood flow (Thal et al., 2008, 2009). CAA in mouse models is described in more detail by Klohs et al. (2014).

Understanding mechanisms by which CAA develops, or plaques deposit on the vasculature, is important in developing AD immunotherapies. Boche et al. (2008) analyzed brains of AD patients that died following immunization against Aβ_42_. Compared to controls, immunized individuals demonstrated more frequent cortical microvascular lesions and microhemorrhages, with significantly more Aβ_42_ deposited on cortical blood vessels and the leptomeninges. Solubilization of Aβ_42_ via immunotherapies may overwhelm perivascular clearance pathways, leading to increased CAA (Greenberg et al., 2020).

Despite similarities between CAA and AD, these diseases are not identical. At least 60 % of individuals above 80 years old are afflicted with CAA, indicating CAA is not restricted to AD (Thal et al., 2008). CAA and AD share additional biomarkers such as cortical thinning and atrophy, cerebrovascular dysfunction, and tau deposition (Greenberg et al., 2020). Advanced CAA is associated with higher levels of cognitive impairment in AD (Greenberg et al., 2020), suggesting a link between aberrant vascular changes induced by CAA and AD development.

Linkages between vascular risk factors and dementia has led AD to be described as a vascular disorder. de la Torre and Mussivand (1993) proposed a hypothetical model where capillaries in brain aging undergo degeneration in response to amyloid deposits. This disturbs blood flow and causes proliferating glia produce APP (de la Torre and Mussivand, 1993; de la Torre, 1994). Factors contributing to brain dysfunction from impaired blood flow include decreased production of energy molecules such as ATP and failure to transmit signals between neurons.

Vascular-based hypotheses have implications for therapeutic targets. Anti-inflammatories, statins, and hypertension drugs are possible alternatives to targeting Aβ (Townsend and Praticò, 2005; Rius-Pérez et al., 2018). Anti-inflammatories reduce Aβ deposition and inflammation in mouse models (Yan et al., 2003; Heneka et al., 2005), with long-term administration of non-steroidal anti-inflammatory drugs lowering AD risk by 30–60% (Herrup, 2010).

### Vascular Dysfunction and Morphology

Examples of abnormal capillary features include pits in vessel walls, loss of vascular innervation by neurons, and tortuous shaped vessels (Scheibel et al., 1989; de la Torre, 1994). An elevated number of non-perfused “string” vessels are present in AD, possibly explaining reduced flow despite occasional similar densities of capillaries between AD and healthy aging (Brown and Thore, 2011; Hunter et al., 2012).

Studies in mice support a role for Aβ in reducing CBF. Administration of Aβ_40_ to cortex in wild type mice reduces cortical blood flow and diminishes CBF increases induced by vasodilators, probably through generation of ROS by Aβ_40_ (Niwa et al., 2000). Mice over-expressing superoxide scavenging enzyme superoxide dismutase-1 (SOD1), or whose cortex was superfused with SOD, reverse vascular deficits induced by amyloid (Iadecola et al., 1999; Niwa et al., 2000). Aβ_42_ did not similarly influence resting CBF or endothelial response to vasodilators, suggesting that Aβ_40_ may have a more significant vascular effect.

A potential mechanism underlying Aβ_40_- and ROS-induced vascular dysfunction was elucidated in Park et al. (2014). Aβ activates innate immunity receptors on endothelial cells, leading to Nox2 production of superoxide. This superoxide reacts with nitric oxide (NO) produced in endothelial cells to produce peroxynitrite (PN). PN damages DNA, leading to a series of reactions that activate calcium-permeable ion channel TRPM2. This permits excessive Ca^2+^ entry into the cell, inducing the observed endothelial dysfunction such as vessel constriction, reduced blood flow, and vascular hyperpermeability. The potential of TRPM2 as a therapeutic target is evaluated further in the Section Discussion.

Endothelial dysfunction does not require plaque accumulation, only exposure to Aβ. This may explain blood flow reductions and observed microvascular abnormalities. Mice overexpressing APP and APP-derived Aβ peptides (Tg2123 and Tg2576) demonstrate blood flow reductions prior to plaque deposition, with the Tg2576 mouseline that possesses higher levels of Aβ demonstrating widespread reductions in CBF (Niwa et al., 2002). This may similarly occur in humans, as early CBF reductions occur prior to neuropathological changes and cerebrovascular responses to functional activation are impaired individuals at high AD risk (Smith et al., 1999; Niwa et al., 2000).

This suggests multiple phases of vascular dysfunction: one occurring prior to vessel loss, the second attributable to vascular destruction and regression. In humans, CBF deficits are present in the pre-dementia phase of AD, while blood volume changes indicative of vascular structural alterations are detected at the AD stage (Lacalle-Aurioles et al., 2014).

In the APP23 mouse model, at young ages prior to plaques, small deposits containing Aβ are found primarily attached to capillaries (Meyer et al., 2008). These deposits, associated with distorted microvessels, are proposed to alter local blood flow. This is believed to trigger increased amyloid production, ultimately resulting in vascular degeneration at older ages (Meyer et al., 2008). Since these deposits contain Aβ_42_, this suggests a role for Aβ_42_ in instigating vascular dysfunction, in addition to Aβ_40_ discussed above. Similar capillary deposits have been observed in humans, lending support to the findings in mice (Miyakawa, 1997).

Studies in the APP/PS1 mouse model suggest capillary dysfunction prior to vessel loss. Despite similar cortical capillary length density in transgenic compared to wild-type mice at 18 months, cortical oxygen availability was reduced in the transgenic mice as indicated by reduced capillary blood flow and elevated capillary transit time heterogeneity at rest (Gutiérrez-Jiménez et al., 2018). This was attributed to factors such as abnormal cholinergic innervation of blood vessels, arterial contraction, or increased vessel tortuosity (Gutiérrez-Jiménez et al., 2018). Compared to wild types, APP/PS1 transgenic mice demonstrate increased blood flow and capillary flow homogenization in response to functional activation (Gutiérrez-Jiménez et al., 2018). The authors note in contrast to humans and other mouse models that display pericyte degeneration, pericyte numbers are not changed in the APP/PS1 model at 18-months. Vasodilation involving pericyte signaling, such as pericyte relaxation at the capillary wall (Hall et al., 2014), may be preserved, explaining the increased flow in response to functional activation (Gutiérrez-Jiménez et al., 2018).

In contrast to early blood flow reductions, some researchers record hyperperfusion in brain regions vulnerable to AD pathology in young, high-risk subjects (Fleisher et al., 2009; Bangen et al., 2012; Østergaard et al., 2013). This may be attributable to early capillary dysfunction that leads to reduced oxygen extraction by tissue from the microvasculature, causing a compensatory increase in CBF (Østergaard et al., 2013, 2015).

### Angiogenesis and Alzheimer's Pathology

Vagnucci and Li (2003) proposed the “Angiogenesis Hypothesis.” They observed drugs which reduce AD risk, such as statins, may possess anti-angiogenic properties. In addition, microvessel density is increased in AD (Perlmutter et al., 1990). In the Angiogenesis Hypothesis, neovascularization occurs in response to reduced blood flow and vascular injury due to inflammation. Pre-amyloid deposits on capillaries generate reactive oxygen species. This causes intravascular accumulation of thrombin and endothelial release of APP.

Studies in the Tg2576 mouse model suggest amyloid could trigger angiogenesis, causing BBB leakage and hypervascularity (Biron et al., 2011). This model demonstrates BBB disruption at 4-months, prior to plaque appearance (Ujiie et al., 2003). Aged (18–24 month old) Tg2576 mice displayed increased cortical microvessel density, similar to humans, corresponding with the degree of tight junction disruption (Biron et al., 2011). The hypervascularity observed in Tg2576 mice was reversed through immunization with Aβ (Biron et al., 2013). In mice, the anti-angiogenic bexarotene (an anti-cancer agent) clears Aβ and restores memory function (Cramer et al., 2012; Jefferies et al., 2013). However, angiogenic activation of endothelial cells occurs following cognitive decline in the APP23 mouse model (Schultheiss et al., 2006), suggesting angiogenesis may not initiate AD.

Some studies contradict the angiogenic properties of amyloid. Most human AD specimens display reduced vessel density (Brown and Thore, 2011). There is reduction in capillary density in white matter in 10-month old Tg2576 mice (Zhang et al., 2019), with Aβ inhibiting angiogenesis (Paris et al., 2004c) and potentially reducing vessel density in cortex and hippocampus (Paris et al., 2004a).

**Figure 1:**
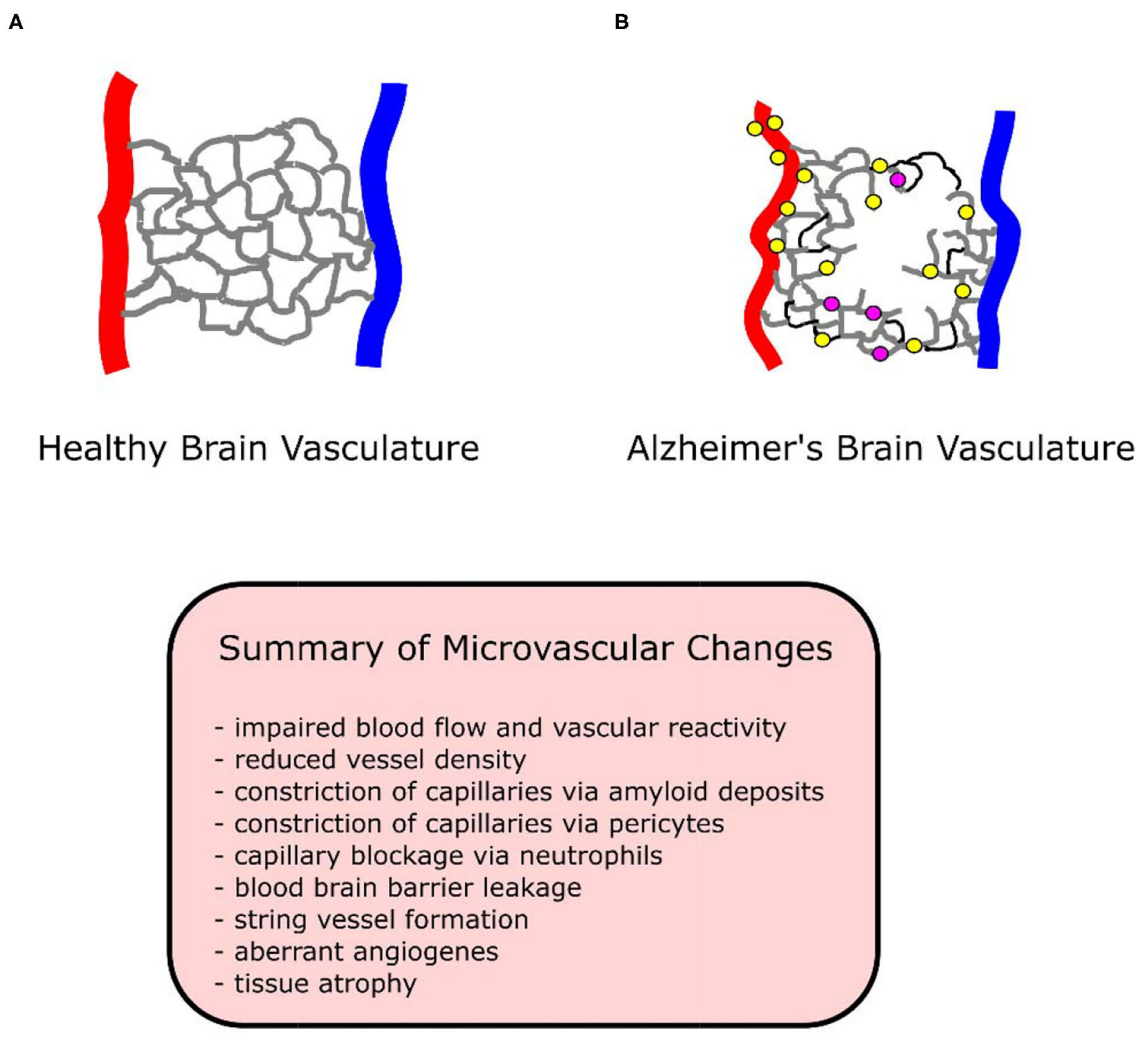
Diagram of healthy and Alzheimer's brain vasculature. **(A)** Diagram of healthy brain vasculature. Penetrating arteries (*red*) and veins (*blue*) are connected via a capillary mesh (*gray*). **(B)** Diagram of Alzheimer's brain vasculature. Deposition of amyloid (*yellow dots*) in the vascular bed causes vascular dysfunction and loss in AD, with proliferation of vessels around missing vasculature (Meyer et al., 2008). String vessels (*black*) with no blood cells form as endothelial cells undergo apoptosis. Neutrophils (*purple dots*) stall a small portion (~2%) of capillaries, reducing blood flow in the network. Penetrating vessels have a tortuous shape, with a tendency to decrease in diameter with aging in AD (Dorr et al., 2012). Capillaries been reported to increase (trending) in diameter in APP/PS1 mice (Lu et al., 2019), experience swelling in APP23 mice (Meyer et al., 2008), decrease in diameter in P301L tau mice (Bennett et al., 2018), and to form “string” vessels with no flow or blood cells in humans (Hunter et al., 2012). Tissue undergoes atrophy, causing vascular shrinkage and functional brain deficits such as memory loss.

There are explanations for these discrepancies. Plaque deposits create “holes” in the vascular bed, while vessels proliferate around these holes (Meyer et al., 2008). An overall measure of vessel density may indicate loss of vessels due to holes, without accounting for the increase in vessels surrounding holes. A simultaneous loss and growth of vessels in human AD specimens has been observed (Desai et al., 2009). Despite the presence of angiogenic vessels in all brain regions, only the hippocampus demonstrated an increase in total capillary density (angiogenic plus non-angiogenic vessels) (Desai et al., 2009). Vessels may reside in a permanent angiogenic state, constantly growing and regressing in human studies (Desai et al., 2009), resulting in no net change or even a loss of vessel density. See Figure 1 for a diagram of vasculature in AD and healthy conditions.

Capillary density in AD models may be dependent on age or brain region. In the Tg2576 mouse model, at 5-months old cortical microvascular density is elevated, while at 27-months density is diminished (Giuliani et al., 2019). In the TgCRND8 mouse line, there is increased microvessel density at postnatal day 7 (Durrant et al., 2020), while in adulthood vessel density is reduced below controls in cortex (Religa et al., 2013). This elevated microvessel density at P7 was accompanied by increased microvessel tortuosity. This may indicate “pathological angiogenesis,” or formation of dysfunctional blood vessels. In the same model (TgCRND8) at 6-months, a decrease in density was found in cortex, while it was increased in hippocampus relative to non-transgenic mice, suggesting a heterogenous angiogenic response depending on brain region (Maliszewska-Cyna et al., 2020).

The reduced vessel density at older ages might be attributable to the aging process and reduced expression of angiogenic growth factors. In brains without AD, decline in growth factors with aging, such as VEGF, fibroblast growth factor (FGF-1 and FGF-2), and angiopoietin, yield slow recovery in tissue wound injuries due to reduced angiogenic capabilities (Ambrose, 2017), or display reduced angiogenesis in response to hypoxia (Benderro and Lamanna, 2011). In a human study, Huang et al. (2013) found significant reduction in serum VEGF relative to both amnestic mild cognitive impairment and control (healthy) individuals. Transforming growth factor β1 (TGF-β1) (an angiogenic growth factor) serum concentration was reduced in AD, with the reduction in VEGF and TGF-β1 levels correlating with cognitive impairment severity (Huang et al., 2013). Huang et al. (2013) hypothesized this indicates reduction in angiogenic growth factors contribute to cognitive impairment. Since VEGF deposits with Aβ, it is possible that Aβ accumulation decreases available VEGF. This leads to reduced growth factor concentration available for angiogenesis, diminishing the number of blood vessels and inducing hypoperfusion (Huang et al., 2013). TGF-β1 reduction in AD patients has been recorded in another study, De Servi et al. (2002) verifying the findings of Huang et al. However, Kim and Kim (2012) recorded AD patients as possessing higher serum VEGF, while angiogenin (protein that stimulates angiogenesis) is reduced. Luppi et al. (2009) recorded significantly reduced VEGF, TGF- β1, and IGF-1 secreted by immune cells in AD patients. Together, these findings provide a potential explanation for reduced vessel density observed in AD.

### Blood Brain Barrier Leakage and Disruption

The BBB is formed through tight junctions between endothelial cells, and regulates movement of ions and molecules between blood vessels and brain tissue. The BBB consists of multiple cell types in addition to endothelial cells, which together comprise the “Neurovascular Unit” (NVU). The cells types of the NVU include endothelial cells, pericytes, smooth muscle cells (arterial and venous), astrocytes, microglia, oligodendrocytes, and neurons (Sweeney et al., 2019).

This protects neural tissue from pathogens or toxins. BBB dysfunction due to brain injury is a risk factor for dementia, resulting in immune cell influx into tissue, perivascular inflammation, CBF alterations, and accumulation of molecules prone to aggregation (Abrahamson and Ikonomovic, 2020).

The BBB is a component of the Two-Hit Vascular Hypothesis (Sagare et al., 2012), where vasculature risk factors reduced blood flow or BBB dysfunction. Flow decreases may be induced by atherosclerosis (Gupta and Iadecola, 2015), while BBB damage could be caused by pericyte degeneration (Miners et al., 2018; Montagne et al., 2018; Wåhlin and Nyberg, 2019). These vascular risk factors and vascular damage are the “First Hit” which initiate Aβ deposition (the “Second Hit”). BBB dysfunction leads to neurotoxin accumulation and impaired Aβ clearance through the BBB. Reduced blood flow due to vascular damage also leads to elevated Aβ production. Together, accumulation of Aβ and hypoxic conditions initiate neuronal dysfunction, degeneration, and loss, causing dementia (Sagare et al., 2012).

BBB damage has consequences in not clearing Aβ and enabling toxins to enter brain tissue. In addition to LRP1 reduction with aging (see Section the Aging Vasculature and Alzheimer's), Receptor for Advanced Glycation End Products (RAGE) on endothelial cells binds circulating Aβ and transports it across the BBB (Deane et al., 2009). RAGE interaction with Aβ contributes to oxidative stress, reduced blood flow, and inflammation (Deane, 2012). BBB leakage causes plasma proteins such as albumin, fibrin, thrombin, and immune cells to enter tissue. These proteins and cells generate neuroinflammation and act on neurons and glia, damaging axons (Strickland, 2018; Klohs, 2019).

Since BBB impairment occurs prior to cognitive impairment or Aβ deposition (Klohs, 2019; Nation et al., 2019), BBB repair is an early target in AD. Due to the connection between neuronal damage, inflammation, and BBB, drugs developed to treat stroke have potential for translation to AD. 3K3A-APC, a drug for treating ischemic stroke, protects BBB through inhibition of endothelial apoptosis (Lazic et al., 2019). In a mouse model of AD administered 3K3A-APC daily for 4 months, neuronal production of Aβ was blocked through inhibition of β-secretase, neuroinflammation suppressed, BBB repaired, and CBF responses to stimulation restored (Lazic et al., 2019). This indicates a role for drugs that simultaneously reduce Aβ production, protect the vasculature, and suppress inflammation.

BBB leakage due to inflammation increases susceptibility to environmental pollutants. Tiny particles absorbed by alveoli circulate through the blood stream, where they may enter the brain, or trigger further inflammation (Peeples, 2020).

### Imaging of Microvascular Dysfunction

One technique extensively applied to image the microvasculature in AD is 2-photon microscopy. 2-photon microscopy uses high intensity, long-wavelength laser light to excite fluorophores close to 1 mm deep into cortical tissue. The vasculature is typically imaged following a tail vein injection of a fluorescent dye. Following imaging, the vasculature is segmented for quantification of structural properties of the network (Steinman et al., 2017, 2019). Blood flow simulations are performed on the vascular segmentations by approximating vessels as tubes (pipes), and application of equations which relate structural properties of the tube (length and diameter) to its blood flow resistance (Schmid et al., 2017).

An example of the benefits of flow simulations is seen in the study by Cruz Hernández et al. (2019), where blood flow reductions were calculated in simulations on mouse and human cortical vasculature due to capillary stalling. Observed flow reductions were attributed to the interconnectivity of the capillary network, where blockage of a single vessel reduces flow in downstream vessels.

Red blood cell (RBC) velocity in individual capillaries may be measured with 2-photon microscopy (Kleinfeld et al., 1998). This was demonstrated by Bennett et al. (2018) in tau P301L transgenic mice. Similar to Cruz Hernández et al. (2019), a number of very small diameter capillaries (<4 um diameter) contained leukocytes adhered to their walls, and flow that was restricted to plasma (i.e., RBCs were absent). These capillaries also demonstrated abnormal morphology, such as a spiral shape.

A metric of capillary network function is the variation in blood flow across the capillary network. In healthy tissue, there is a wide distribution of RBC velocities in capillaries, resulting in reduced oxygen extraction fraction (OEF) from blood vessels by tissues (Kleinfeld et al., 1998; Jespersen and Østergaard, 2012; Østergaard et al., 2015). In contrast, a more homogeneous RBC flux across capillaries and capillary oxygenation yields elevated OEF, which occurs in the case of neuronal stimulation (Li B. et al., 2019). Capillary flow patterns are affected by pericytes, dimensions of cells passing through capillaries, glycocalyx, blood composition, and adhesion of cells on vessel walls (Østergaard et al., 2015).

Capillary transit time (the time required for blood to pass through the capillary network) may be observed with 2-photon microscopy by monitoring the passage of a fluorescent dye through the network. A 2-photon study of the TgCRND8 mouse model demonstrated increased transit time dispersion (variability), together with increased transit time, indicative of decreased microvascular network efficiency (Dorr et al., 2012). Transit time dispersion was reduced to non-transgenic levels by treatment with scyllo-inositol, which reduces soluble and insoluble Aβ levels. Since Aβ damages endothelial cells, pericytes, and induces oxidative stress, removal of Aβ-induced endothelial dysfunction was interpreted as the reason for return to normal levels. These mice demonstrated an impaired hypercapnic response, which has been observed in both capillaries and arterioles in a transgenic rat model with amyloid and tau pathology (Joo et al., 2017). Impaired homogenization of transit times has been observed in the APP/PS1 mouse model in response to functional activation (Gutiérrez-Jiménez et al., 2018).

2-photon microscopy is not applicable to human studies as it requires invasive surgical procedures such as a craniotomy. Nielsen et al. (2017) performed perfusion MRI to calculate capillary transit time heterogeneity and mean transit time. Elevated mean transit time and capillary transit time heterogeneity in AD patients correlated with cortical thinning and poor cognitive performance. This study indicated a role for capillary dysfunction in contributing to cognitive decline in humans. Parameters measured in this study included CBF, microvascular cerebral blood volume, and tissue oxygen tension, whose reduction also correlated with poor cognitive performance (Nielsen et al., 2017).

In addition to transit time heterogeneity, MRI techniques (such as dynamic contrast enhanced MRI) may be used to evaluate BBB permeability (Raja et al., 2018).

The vascular changes discussed for the main mice models in this section (Section Vasculature in Alzheimer's Disease) are summarized in a Table 1 below.

**Table 1:** Summary of cerebral vascular changes in the main mouse models highlighted.

**Mouseline**	**Vascular changes**
TgCRND8	- Elevated cortical vessel density at P7 and increased microvascular tortuosity (Durrant et al., 2020)
	- Reduced cortical vascular density, increased hippocampal vessel density at 6 months (Maliszewska-Cyna et al., 2020). - Increased hippocampal non-capillary (arterial/venule) tortuosity (Maliszewska-Cyna et al., 2020).
	- Increased cortical penetrating arteriole tortuosity due to amyloid deposition. Longer transit time (relative to controls) and transit time variability. Hypercapnia results in transit time increase. No change in tortuosity of penetrating venules (Dorr et al., 2012; Lai et al., 2015)
Tg2576	- Widespread reductions in CBF at 2–3 months (Niwa et al., 2002)
	- BBB disruption at 4 months prior to plaques (Ujiie et al., 2003)
	- Increased cortical capillary density at 5 months when memory impaired and plaques not formed; capillary density diminished by 27 months (Giuliani et al., 2019)
	- Capillary density reduction in white matter at 10 months (Zhang et al., 2019)
APP/PS1	- Cerebral capillaries possess microaneurysms along their lengths, possible BBB breakdown, at 4–5 months prior to memory deficits; elevated cortical plaque number at this time point. No change in capillary diameter or length (Kelly et al., 2017)
	- No change in vessel density by 18 months, but elevated capillary transit time heterogeneity and reduced capillary blood flow at rest. Compared to wild types, demonstrate increased blood flow and capillary flow homogenization in response to functional activation (Gutiérrez-Jiménez et al., 2018)
APP23	- At young ages prior to plaques (4–6 months), deposits containing amyloid attach to capillaries; are associated with distorted blood vessels. The number and size of holes in the vascular bed increases. There are indicators of angiogenesis surrounding the holes (Meyer et al., 2008)
	- By 25–26 months, CAA-associated capillary occlusions in thalamus cause abnormal blood flow in perforating arteries (Thal et al., 2009)

## Discussion and Concluding Thoughts

Many research issues are unresolved. This includes, for example, the contributions of the various types of Aβ (monomers, soluble oligomers, and plaques/fibrillar Aβ) to AD (Hillen, 2019), and understanding why amyloid is unsuccessfully cleared and which mechanisms to clear plaques fail in a particular individual.

The AH was reviewed, including explanations for failures. Pathological mechanisms beyond amyloid were outlined. Interdisciplinary studies involving microvascular imaging and modeling in mouse models were highlighted, demonstrating microvascular malfunctions that exacerbate AD.

Despite promise in exploring alternative mechanisms, it is premature to abandon the AH. As noted in Walsh and Selkoe (2020), additional analyses of the Phase 3 aducanumab trials indicated that in one trial, there was an “~40% less decline in activities of daily living than occurred on placebo.” With another antibody (BAN2401) demonstrating success in a Phase 2b clinical trial (Walsh and Selkoe, 2020), there are indications that anti-Aβ therapies could reach a breakthrough.

It is important to investigate alternate avenues by which AD develops beyond Aβ. This affects strategies and approaches for designing research activities and development of treatments and drugs. For example, when plaques and tangles are both present, eliminating each simultaneously might be a preferred option rather than amyloid only. If neuroinflammation is widespread, clearing amyloid without altering the inflammatory state may not reduce cognitive deficits.

There are numerous AD hypotheses in addition to Aβ, yet it is difficult to determine the relevancy of each hypothesis, since AD develops according to genetic, sex, and environmental factors that differ for each subject. AD could present in a variety of forms, with plaques and tangles in common (Frenkel, 2020). For example, atherosclerosis or cardiac dysfunction may appear in one AD patient, while be absent in another. This impacts the design of effective treatments since optimal therapies possibly should be “personalized” according to an individual's age, genetics, risk factors, and timing of diagnosis. Frenkel (2020) noted there is the potential of an “arsenal of drugs that can be used to personalize medicine for each patient in the spectrum of diseases associated with AD.” This approach faces challenges, such as determining which “cocktail” of drugs to use and how drugs interact with one another.

Below are two approaches to treat AD with targeting Aβ: VEGF therapy and TRPM2 inhibition.

### Non-amyloid Treatments

#### VEGF and Growth Factor Therapy

Due to reduced capillary density with aging, (Ambrose, 2012) proposed VEGF and growth factor administration for restoring capillary density and blood flow. A study in TgCRND8 AD mice overexpressing VEGF found partial recovery of vessel density and restoration of memory impairments, supporting enhancing vascular growth as a method for improving cognition (Religa et al., 2013).

There are factors to consider in applying this therapy to humans. VEGF in high concentrations may induce BBB leakage. Many newly formed vessels during angiogenesis are leaky with abnormal morphology (Vogel et al., 2004). Increasing vessel density alone does not necessarily elevate blood flow (Vogel et al., 2004). In VEGF-overexpressing mice subject to middle cerebral artery occlusion, flow is increased in regions outside the ischemic zone, but reduced within the ischemic/injured tissue core (Wang et al., 2005).

Ambrose (2012) reviews multiple methods for administering growth factors based on animal studies. These include: (1) intravenous delivery via a pump placed under the skin; (2) injection into CSF in ventricles; (3) injection into subarachnoid space above the cerebral hemispheres; (4) magnetic particles loaded with angiogenic factors that are held in the brain via a magnetic skullcap; (5) continuous release of growth factors via direct insertion of a growth factor releasing material into the brain; (6) surgical insertion (i.e., drilling holes in skull) of growth factor-loaded biodegradable material over cortex; (7) injection of growth factors into nasal cavity.

These delivery methods are complicated. For example, a pump placed under the skin would deliver VEGF throughout the body, not only the brain (Ambrose, 2012). Methods involving surgery (injection into CSF, drilling holes in skull) are invasive. A major issue is the growth factors to be delivered. Regrowth of vascular is more than increasing vessel number through administering VEGF. Newly formed vessels adjust their diameters, potentially differentiate into arteries or veins, and recruit support cells such as smooth muscle cells, pericytes, and fibroblasts to produce a functional vessel network (Gale and Yancopoulos, 1999). This network possesses optimal blood flow to satisfy tissue metabolic requirements, and may increase flow through adjusting diameters in response to functional activation of tissue. VEGF stimulates endothelial cell division and migration to form new vessels. Angiopoietin 1 (Ang1) facilitates communication between endothelial and support cells such as smooth muscle cells, stabilizing vessels and acting in conjunction with VEGF to produce a functional network (Gale and Yancopoulos, 1999). Delivery of a single growth factor, such as VEGF, risks producing vessel networks prone to leaks or hemorrhage (Yancopoulos et al., 2000). Expanding growth factor therapy to AD will require consideration of relative concentrations of naturally occurring growth factors unique to each subject and state of dementia, delivery method, and determining growth factors to administer (i.e., VEGF, Ang1 or 2, FGF, ephrins, etc.), quantity/concentration of growth factors to deliver, and length of time of treatment (i.e., single dose, multiple low-level doses, etc.).

A related therapy to growth factor administration that overcomes some deficiencies is exercise. Exercise increases concentration of a variety of angiogenic molecules such as Ang1 and 2, VEGF, fibroblast growth factor (FGF, upregulates VEGF, and induces vasodilation through nitric oxide), transforming growth factor (TGF, regulates extracellular matrix formation), and platelet derived growth factor (PDGF, mitogen for smooth muscle cells, fibroblasts, and glia cells) (Korivi et al., 2010). Mice provided access to running wheels demonstrated elevated brain microvascular efficiency (Dorr et al., 2017) and increased blood flow in the hippocampus (Cahill et al., 2017). In APP/PS1 mice, exercise restored vessel density and decreased capillary flow heterogeneity in cortex, likely indicating increased oxygen delivery (Lu et al., 2019). Exercise in TgCRND8 mice restored normal capillary density in cortex and hippocampus, and restored hippocampal arterial/venular tortuosity in AD mice to control levels (Maliszewska-Cyna et al., 2020). Since exercise did not alter parenchymal or vascular amyloid levels, improvements in short-term are possibly attributable to these vascular changes (Maliszewska-Cyna et al., 2020).

#### Inhibition of Transient Receptor Potential Melastatin 2 (TRPM2)

TRPM2 is a calcium-permeable ion channel expressed in multiple organs and cell types, including the brain (Turlova et al., 2018). It is activated in response to reactive oxygen species and oxidative stress, permitting calcium to enter the cell and causing neuronal death if the calcium concentration is sufficiently high (Turlova et al., 2018). Our labs demonstrated that eliminating TRPM2 expression or activity in neonatal hypoxic-ischemic injury improves behavioral performance and reduces tissue damage (Huang et al., 2017; Li F. et al., 2019).

Inhibition of TRPM2 activity demonstrates potential in AD. Ostapchenko et al. (2015) demonstrated that APP/PS1 TRPM2^−/−^ mice display reduced microglial activation, reversal of memory deficits, and decreased synapse loss. Plaque load or soluble Aβ peptides were not altered, indicating potential for recovery of cognitive function without directly affecting plaque concentration (Ostapchenko et al., 2015).

Activated TRPM2 damages tissue through several mechanisms. It promotes inflammation through cytokine production, and induces BBB dysfunction by allowing calcium into endothelial cells in response to oxidative stress (Belrose and Jackson, 2018). As previously mentioned, Aβ may cause endothelial and vascular dysfunction (such as arterial constriction) by increasing oxidative stress, activating a DNA repair enzyme that opens endothelial TRPM2 channels (Park et al., 2014). Since inhibiting or genetically deleting TRPM2 restores neural activity-induced CBF increases (Park et al., 2014), targeting TRPM2 has potential to restore cerebral vascular function in AD.

Potential mechanisms of TRPM2 action are highlighted in cell culture studies. In human pulmonary artery endothelial cell cultures, inhibition of TRPM2 via small interfering RNA decreased H_2_O_2_-induced intracellular calcium and the consequent increase in endothelial permeability (Hecquet et al., 2008). Ca^2+^ entry via TRPM2 may also initiate a process inducing endothelial dysfunction or apoptosis through actions on mitochondria. In cultured pancreatic β-cells in culture, increase in ROS activates TRPM2, causing Ca^2+^ cytoplasmic entry and Zn^2+^ release from lysosomes. Zn^2+^ binds to mitochondria, inhibiting the electron transport chain and leading to membrane potential loss that recruits dynamin-related protein1 (Drp1). Drp1 “slices” the mitochondria, causing mitochondrial fission and apoptosis (Li et al., 2017a,b). A similar process occurs in cultures of endothelial cells in response to increased ROS (Abuarab et al., 2017), with hippocampal neurons exposed to Aβ also demonstrating zinc accumulation in mitochondria and mitochondrial dysfunction (Li and Jiang, 2018).

Scalaradial, a marine extract, is a potent TRPM2 inhibitor, although it partially inhibits TRPM7 as well (Starkus et al., 2017). Its mechanism is unknown. Generally, while TRPM2 inhibitors exist, they lack potency or are non-specific (Starkus et al., 2017).

### Looking Ahead and Thoughts

Restoring blood flow in early stages of AD progression could limit dementia progression. Korte et al. (2020) outline additional therapeutic approaches targeting blood flow. Capillary constriction via pericytes occurs via Aβ-oligomer production of ROS from pericytes and microglia and activation of endothelin A receptors that bind endothelin (a vasoconstrictor). Relaxing pericytes via voltage-gated calcium channel (VGCC) inhibition is a therapeutic target, with a VGCC-inhibitor (nilvadipine, normally used for hypertension) restoring cortical CBF in 13-month old Tg2576 mice, and hippocampal CBF in humans with mild-moderate AD (Paris et al., 2004b; de Jong et al., 2019; Korte et al., 2020). Additional therapies suggested by Korte et al. (2020) include preventing occluded capillaries through neutrophil targeting and anticoagulants to enhance blood flow. However, depletion of neutrophils may lead to infection, while anticlotting agents could induce hemorrhage (Korte et al., 2020).

A limitation in developing vascular-based drugs is animal models. A 3D microscopy analysis revealed amyloid plaques in human tissue were larger and more complex than those in mice, displaying a wider variety of sizes and shapes compared to those in mice (Liebmann et al., 2016). Consequently, successful removal of plaques from mice does not necessarily correspond to an identical situation in humans. Other limitations of transgenic rodent models are detailed in Braidy et al. (2012) such as difficulties in modeling tau pathology and lack of neurodegeneration. *In vitro* models, while valuable for investigating molecular mechanisms, face limits in translation to humans due to inability to account for multiple cellular interactions that affect endothelial cells. Progress has been made on this front through development of a 3D *in vitro* microfluidic culture model containing neurons (developed from progenitor cells) with mutations in APP and APP/PS1 genes, aggregation of extracellular Aβ, hyperphosphorylated tau, and mimicking BBB disruption with aggregation of Aβ on vascular wall (Choi et al., 2014; Kim et al., 2015; Shin et al., 2019). This culture model could be used to screen whether drugs cross the BBB and their effect on different cell types (Shin et al., 2019).

Despite our vascular focus, AD pathology may be improved through treatments that induce negative vascular effects. For example, nilotinib, a tyrosine kinase inhibitor normally used to treat leukemia, was administered to individuals with AD mild to moderate dementia in a phase 2 clinical trial (Turner et al., 2020). Nilotinib reduced CSF Aβ (40 and 42), minimized hippocampal volume loss, and reduced frontal lobe amyloid (Turner et al., 2020). However, nilotinib inhibits endothelial cell proliferation in culture, while inhibiting angiogenesis and blood flow following hind-limb ischemia in mice (Hadzijusufovic et al., 2017). Arterial occlusive disease developed in nilotinib-treated patients, while atherosclerosis and a pro-atherogenic phenotype was promoted in mice models and endothelial cultures respectively (Hadzijusufovic et al., 2017).

Early AD detection is necessary to deliver treatment prior to permanent tissue damage. Kosik (2013) proposed observing activity of collections of neurons, which may show subtle alterations not detectable with standard neuropsychological testing. Such changes could be detectable with resting state functional MRI to monitor connectivity between brain regions (Brier et al., 2014). This is supported by findings in mice, where regional neuronal activity determines location of Aβ deposition (Bero et al., 2011). Some studies note that in humans reduced blood flow precedes cognitive decline (Iturria-Medina et al., 2016), with impaired reactivity to hypercapnia a biomarker of disease (Glodzik et al., 2013).

It is necessary to consider multiple avenues by which AD develops beyond strictly Aβ in developing pharmacological approaches. The AH remains prominent, although reservations have been raised as to its underlying assumption of amyloid as the central feature of AD pathology. As research continues testing more hypotheses, a continued flexibility in approach is required beyond the belief that eliminating plaques/Aβ alone will lead to a cure.

A single treatment approach could be considered for early onset AD since this form of the disease is often dependent on APP-related genetics. Most cases, however, are late-onset and depend on genetic elements beyond Aβ. Consideration should be given to treating multiple mechanisms simultaneously, such as removing plaques through antibodies and reducing inflammation through non-steroidal anti-inflammatories. This approach does not entail abandoning Aβ, but viewing it as an important element of AD pathology that acts in combination with other mechanisms.

To target multiple sites, there are issues to be addressed. For example, is amyloid build-up due to decreased LRP1 expression at the blood brain barrier, reduced neprilysin production, or the glymphatic system and arterial stiffening? And how can these be detected through imaging or biomarkers? To address these issues, the investigator requires understanding the biological mechanisms involved in disease progression, and consequences of therapeutic targeting of these mechanisms while omitting others.

Future research will continue to focus on the many issues covered in this review. Hopefully, this will lead to new research directions and treatments, and improve or prevent cognitive decline in patients.

## Author Contributions

All authors listed have made a substantial, direct and intellectual contribution to the work, and approved it for publication.

## Conflict of Interest

The authors declare that the research was conducted in the absence of any commercial or financial relationships that could be construed as a potential conflict of interest.
